# Comparing conventional and modified Seldinger techniques using a micro-insertion kit for PICC placement in neonates: a retrospective cohort study

**DOI:** 10.3389/fped.2024.1395395

**Published:** 2024-05-02

**Authors:** Matheus F. P. T. van Rens, Kevin Hugill, Robin van der Lee, Airene L. V. Francia, Fredericus H. J. van Loon, Mohammad A. A. Bayoumi

**Affiliations:** ^1^ Neonatal Intensive Care Unit, Radboud University Medical Center, Amalia Children's Hospital, Nijmegen, Netherlands; ^2^ Neonatal Intensive Care Unit, Women's Wellness and Research Center, Hamad Medical Corporation, Doha, Qatar; ^3^ Department of Nursing and Midwifery Education, Hamad Medical Corporation, Doha, Qatar; ^4^ Anesthesiology, Intensive Care and Pain Medicine, Catharina Hospital, Eindhoven, Netherlands; ^5^ Faculty of PeriOperative Care & Technology, Institute of People and Health Sciences, Fontys University of Applied Sciences, Eindhoven, Netherlands

**Keywords:** neonate, neonatal intensive care unit (NICU), vascular catheters, modified Seldinger technique (MST), peripherally inserted central catheter (PICC), complications, newborn, central line-associated blood stream infection (CLABSI)

## Abstract

**Objective:**

This study aims to assess the comparative effectiveness of a conventional splitting needle or a peelable cannula vs. the modified Seldinger technique (MST) by utilizing a dedicated micro-insertion kit across various clinically significant metrics, including insertion success, complications, and catheter-related infections.

**Methods:**

We conducted a retrospective observational cohort study using an anonymized data set spanning 3 years (2017–2019) in a large tertiary-level neonatal intensive care unit in Qatar.

**Results:**

A total of 1,445 peripherally inserted central catheter (PICC) insertion procedures were included in the analysis, of which 1,285 (89%) were successful. The primary indication for insertion was mainly determined by the planned therapy duration, with the saphenous vein being the most frequently selected blood vessel. The patients exposed to MST were generally younger (7 ± 15 days vs. 11 ± 26 days), but exhibited similar mean weights and gestational ages. Although not statistically significant, the MST demonstrated slightly higher overall and first-attempt insertion success rates compared to conventional methods (91 vs. 88%). However, patients undergoing conventional insertion techniques experienced a greater incidence of catheter-related complications (*p* < 0.001). There were 39 cases of catheter-related bloodstream infections (CLABSI) in the conventional group (3.45/1,000 catheter days) and eight cases in the MST group (1.06/1,000 catheter days), indicating a statistically significant difference (*p* < 0.001). Throughout the study period, there was a noticeable shift toward the utilization of the MST kit for PICC insertions.

**Conclusion:**

The study underscores the viability of MST facilitated by an all-in-one micro kit for neonatal PICC insertion. Utilized by adept and trained inserters, this approach is associated with improved first-attempt success rates, decreased catheter-related complications, and fewer incidences of CLABSI. However, while these findings are promising, it is imperative to recognize potential confounding factors. Therefore, additional prospective multicenter studies are recommended to substantiate these results and ascertain the comprehensive benefits of employing the all-in-one kit.

## Introduction

Peripherally inserted central catheters (PICC) are routinely used for sick term and preterm neonates. These devices can be conveniently inserted percutaneously at the bedside and represent a sizable proportion of the central vascular access devices (CVAD) used for vascular access (VA) in neonatal intensive care units (NICU) (1–5). Typically, they are inserted to provide reliable intravenous access for prolonged therapy durations and parenteral nutrition or for infusion therapy in cases of difficult intravenous vascular access (DIVA) (1, 4–6). PICCs used in these circumstances are reported to have lower complication rates compared to short peripheral IV catheters (PIVC) or umbilical venous catheters (UVC) (1, 6–13).

Recognizing PICC candidates early, having an experienced inserter with a developed understanding of neonatal anatomy, and choosing the optimal vein using technological aids for vessel selection and catheter tip placement all help increase the likelihood of a successful placement (6, 7, 13, 14–16). However, PICC insertion is often complicated by the neonate's small size, the fragility of their blood vessels, and previous use of peripheral veins for PIVC use (6, 7, 10, 12). Traditionally, in neonates, PICCs were inserted using a combination of split needle or peelable cannula techniques (5, 16). This approach is supported by a range of commercially available and dedicated medical products and remains a popular choice among many clinicians.

The modified Seldinger technique (MST) is a development of the classic Seldinger technique. The classic Seldinger technique, which was named after its developer and exponent, typically involves several distinct steps. The first step is the needle puncture of the target blood vessel and then the insertion and threading of a flexible guidewire to the estimated final tip location, followed by the removal of the puncture needle. Next is the dilation of the vessel using a dilator before passing the catheter over the guidewire to its intended location. The final step is the removal of the guidewire leaving the catheter *in situ*. The MST is subtly different from the classic technique in its steps. It was developed to take advantage of the advances in VA equipment design and address some of the shortcomings of the original technique (6, 7).

The MST is used for the minimally invasive percutaneous placement of CVCs with the assistance of a guidewire inserted into a suitable dilated peripheral blood vessel (6, 7, 16, 17). Typically, the technique involves a needlestick with a puncture needle, followed by the insertion of a short guidewire. The needle is then removed. Using a guidewire and an insertion aid, a combined dilator and peelable cannula are inserted over the guidewire into the blood vessel lumen. The guidewire and insertion aid are then removed. The catheter is fed through the dilator/cannula and threaded to its desired tip position before the dilator/cannula itself is withdrawn and peeled apart to separate it from the catheter for disposal (6, 7, 16–20). Catheter stabilization, securement, dressing, and confirmation of the correct catheter tip location using medical technologies are similar between the two approaches (6, 7).

The MST has been widely adopted in pediatric and adult populations, but minimum blood vessel diameters precluded its use in smaller patients. In recent years, the technological advances in VA device design, particularly with smaller sizes becoming available, have led to this technique being used in NICUs (16). More recently, all-in-one MST kits with micro components suitable for use with neonates have been made commercially available. These kits contain matched micro bore needles, vein dilators, and guidewires supporting the insertion of neonatal-size PICCs into smaller, more superficial peripheral veins while potentially decreasing venous trauma and enhancing first-attempt insertion success rates (4, 16–20). In this article, for clarity, we refer to proprietary all-in-one micro-component MST kits as “micro-MST kit.” This phrasing acknowledges the neonate compatible size of its components and relationship to the MST insertion technique and differentiates this kit from more traditional *ad hoc* combinations of equipment for MST.

To date, there are few comparative reports on the use of micro-sized MST kits or factors affecting their use in neonatal populations (14). In this article, we present a study that evaluates the MST using the micro-MST kit against conventional PICC insertion techniques (split needle or peelable cannula). The measures of insertion success, therapy completion or failure (necessitating early unplanned catheter removal), and infection rates, which are clinically important outcomes affecting the overall success or failure of IV therapy, are reported herein.

## Materials and methods

A retrospective observational cohort study design was used. The study objective was to evaluate different methods of PICC insertion (i.e., conventional: steel splitting needle or peelable cannula and a modified micro-Seldinger technique insertion kit) in NICU neonates to identify the most effective technique for reducing complication rates (specifically, first-attempt insertion success, therapy completion, and infection) among the study population. The primary outcome measure consisted of the successful insertion of a PICC, encompassing both the completion of the insertion procedure itself and the accurate positioning of the catheter tip in accordance with international guidelines (6, 7). The process measures involved the percentage reaching the end of therapy without related complications. The study protocol was approved by an institutional review board (MRC-01-22-626).

### PICC insertion

Historically, in our unit, all PICCs, except those surgically inserted, were inserted using either a steel splitting needle or peelable cannula, each manufactured/distributed by the medical equipment company, Vygon:

• Microflash, a 20G peelable cannula introducer for 1 and 2Fr PICCs
• Siliconized stainless-steel 24G Splitting needle for the insertion of 1Fr catheters

**Figure 1:**
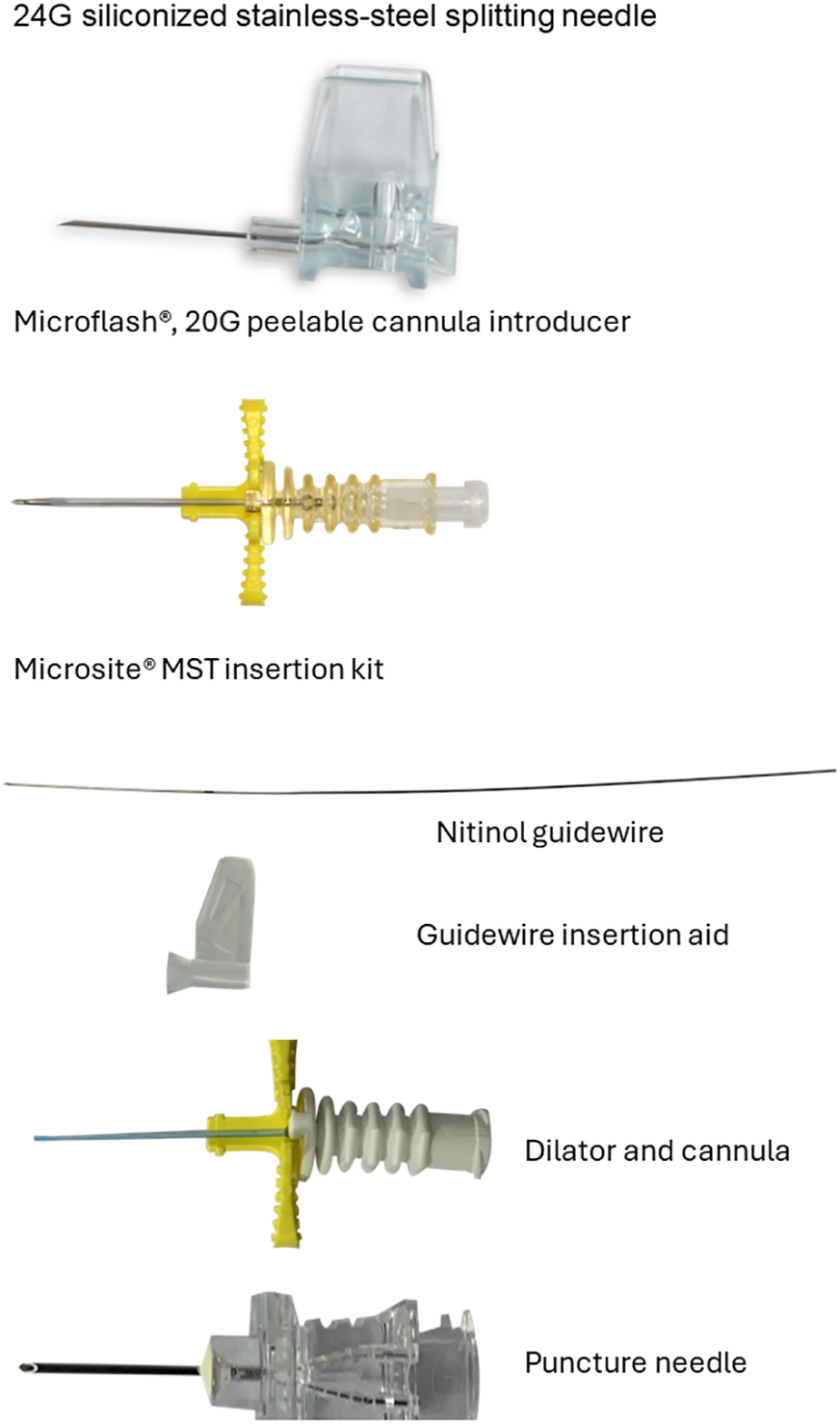
Peripherally inserted central catheter (PICC) insertion devices. Images courtesy of and used with permission from Vygon, France.

A review of internal audit data showed a high number of complications related to the stainless-steel splitting needle technique, including infusate leakage, puncture, and breakage of catheters. While the peelable cannula technique had fewer insertion-related issues, the first-attempt success rates were lower. This observation was felt to reflect the clinician’s skill level, familiarity with a particular technique, and lack of standardized training. With the formal establishment of a multi-professional neonatal VA team (NeoVAT), this situation changed (14). This team was exposed to evidence-based training and education based on international standards and care bundles (6, 7, 15) and a standardized confirmation of competence assessment. They now provide a dedicated VA service (14). In 2017, following a period of orientation and training, the micro-MST using a Vygon Microsite insertion kit containing a 24G puncture needle, a flexible nitinol guidewire, a guidewire insertion aid, and a 20G peelable combined dilator/cannula was introduced as an option for PICC insertion. Figure 1 illustrates the three PICC insertion devices.

In the study unit, MST using the micro-MST approach follows the established evidence-based standard procedures (6, 7, 15) outlined in the Introduction section and described previously (16). For readers less familiar with neonatal PICC insertion using the MST, short audio–visual presentations are readily available, for example, https://m.youtube.com/watch?v=z4zcmY1DrVE and https://vimeo.com/478943739, which show the steps involved.

During the initial patient assessment, the NeoVAT uses a locally developed mnemonic—the “5 Rights for Vascular Access”—to ensure the selection of the right device for the right vein when administering the right therapy for the right duration and for the right patient (4, 15, 21). This systematic approach used in in-house training programs helped maintain a standardized and patient-centric practice during VA. Evidence-based care bundles of preventative infection control measures for insertion site dressing and dressing changes, catheter-related bloodstream infections (CLABSI) prevention together with a daily consideration of the need for continued VA, and planned removal are implemented and routinely audited.

### Data

Routinely collected anonymized VA data were collected between 1 January 2017 and 31 December 2019. The study was carried out in the NICU of the Women's Wellness and Research Centre (WWRC) of the Hamad Medical Corporation (HMC, Doha, Qatar). The data collected were sex, Gestational age (GA) at birth, age at time of insertion, birth weight, reason for insertion, vein used, technique used for insertion, success of insertion, dwell time, reason for removal, and presence of infection.

### Participants and sample size

The study site was a large 112-bed tertiary-level NICU with approximately 4,000 admissions yearly. Approximately 500 PICCs are inserted each year. The study population included all neonates admitted to the NICU requiring a PICC for IV fluids, parenteral nutrition, administration of medications, or DIVA defined as requiring more than three PIVCs in 24 h.

Neonates were excluded from the data if VA devices other than PICCs were used (e.g., PIVCs and umbilical or surgically inserted central catheters). To ensure a maximal set of data and the inclusion of all eligible participants, the NeoVAT members checked patient charts each day for any omissions.

### Statistical analysis

The data were analyzed based on the PICC insertion method and the device used. The analysis of the patient characteristics and outcome variables was summarized using descriptive measures expressed as mean (standard deviation) or median (minimum–maximum) for continuous variables and frequencies expressed as percentages for categorical variables. The assumption of normal distribution was determined with Kolmogorov–Smirnov testing. To detect the significance between dependent and independent variables, Chi-squared test, unpaired *t*-test, Mann–Whitney *U* test, or one-way analysis of variance was used, as appropriate. *Post hoc* analyses [Tukey honestly significant difference (HSD) test] were used to detect the differences between subgroups. SPSS (version 27.0) was used for all statistical analyses, with *p* < 0.05 representing the level of significance.

## Results

### Overview

Between 1 January 2017 and 31 December 2019, a total of 1,445 insertion procedures were recorded (Table 1). The patient characteristics were reported for all 1,445 neonates. In addition, data on catheter characteristics, complication rates, associated factors for complications, and number of insertion attempts are reported.

**Table 1:** Baseline demographic characteristics of the study participants.

		Conventional insertion (*n* = 887)	Micro-MST (*n* = 558)	*p-*value
Sex				
Male	792	479 (54%)	313 (56%)	0.437
Female	653	408 (46%)	245 (44%)	
Age (days) at insertion, mean ± SD		11 ± 26	7 ± 15	0.001
Gestational age (GA) at birth (weeks)				
23–27	359	222 (25%)	137 (25%)	
28–31	655	412 (47%)	243 (44%)	
32–36	224	110 (12%)	114 (20%)	
≥37	207	143 (16%)	64 (11%)	
GA mean ± SD (weeks)		29.5 ± 3.9	29.8 ± 3.9	0.051
Birth weight (g)				
≤999	504	342 (39%)	162 (29%)	<0.001
1,000–1,499	632	383 (43%)	249 (45%)	
1,500–2,499	202	90 (10%)	112 (20%)	
≥2,500	107	72 (8%)	35 (6%)	
Birth weight, mean ± SD		1,269 ± 639	1,344 ± 681	

SD, standard deviation.

The mean birth weight was 1,269 g (±639) in the conventional insertion group and 1,344 g (±681) in the micro-MST group. The mean GA was 29.5 (±3.9) weeks in the conventional insertion group and 29.8 (±3.9) weeks in the micro-MST group. The most common diagnosis for admission was prematurity.

In Table 2, the reasons for the PICC insertion and the specifics regarding the fluids and DIVA are presented. The larger majority of the inserted PICCs were used for prolonged intravenous therapy either for parental nutrition and/or medication. The patient characteristics were mainly related to weight [≤1,500 g, as per local protocols (15, 20, 22)] and difficult intravenous access. The fluid characteristics of the parental nutrition or related medication included a pH of <5 or >9 and an osmolarity of >600 mOsm/L as per local protocols (16, 21, 23). The analysis of the choice of the “vein inserted” data indicated a difference between the conventional and micro-MST study groups in terms of the selected puncture site/inserted vein (*p* = 0.011). The lower limbs were targeted most often for insertion, and the most common vein used for both groups was the saphenous vein (67% for conventional and 76% for micro-MST).

**Table 2:** Reason for the peripherally inserted central catheter (PICC) insertion and targeted blood vessel.

Reason for insertion	Total *n* = 1,445	Conventional insertion (*n* = 887)	Micro-MST (*n* = 558)	*p-*value
Duration of therapy	1,368	865 (97.5%)	503 (90.1%)	<0.001
Fluid characteristics^a^	27	7 (0.8%)	20 (3.6%)	
Patient characteristics^b^	50	15 (1.7%)	35 (6.3%)	
Targeted blood vessel				
Limb extremity inserted				
Upper	421	292 (33%)	129 (23%)	<0.001
Lower	1,024	595 (67%)	429 (77%)	
Vein inserted				
Axillary	2	1 (0.1%)	1 (0.2%)	0.011
Basilic	34	21 (2.4%)	13 (2.3%)	
Brachial	10	8 (0.9%)	2 (0.4%)	
Cephalic	56	39 (4.4%)	17 (3.0%)	
Cubital	310	216 (24.4%)	94 (16.8%)	
Dorsal	7	5 (0.6%)	2 (0.4%)	
Femoral	1	0 (−)	1 (0.2%)	
Jugular	2	2 (0.2%)	0 (−)	
Saphenous	1,023	595 (67.0%)	428 (76.7%)	

[After, (15, 20, 22)].

^a^ pH (<5 or >9) and/or osmolarity (>600 mOsm/L).

^b^ Weight (≤1,500 g) and/or difficult intravenous vascular access (DIVA) (>3 peripheral IV within 24 h).

### PICC insertion success by method and device

In a total of 1,445 insertion procedures, 1,286 catheters (89%) were successfully inserted (Table 3). Table 3 shows the insertion success of the individual devices as per group (i.e., micro-MST and conventional) and as per insertion device type (i.e., Microsite, Microflash, and Splitting needle). The success rate was slightly higher for MST (91% success vs. 88%). The difference increased when analyzing the used insertion needle type. Microsite used micro-MST as an insertion technique and showed significant differences with either Microflash or the Splitting needle. The same was observed when analyzing the number of attempts to one successful insertion, that is, micro-MST showed a significantly higher success rate.

**Table 3:** Peripherally inserted central catheter (PICC) insertion success by method and device.

		Conventional insertion (*n* = 887 attempts)		Micro-MST (*n* = 558 attempts)	*p-*value
Successful insertions	1,286 (89%)	781 (88%)		505 (91%)	0.130
Number of attempts of successful insertion (median and IQR)		1.7 1 (1–6)		1.4 1 (1–4)	<0.001
		Conventional insertion		Micro-MST	
		Microflash *n* = 485	Splitting needle *n* = 403	Microsite *n* = 558	
Successful insertions		432 (89%)	349 (87%)	505 (91%)	0.163
Number of attempts of successful insertion (IQR)		1 (1–5)	1 (1–6)	1 (1–4)	

IQR, interquartile range.

The use of the Microsite device resulted in the highest first-attempt success rate of insertion when compared to conventional Microflash and Splitting needle. *Post hoc* analyses resulted in the significance between Microsite and Microflash (*p* < 0.001), Microsite and Splitting needle (*p* = 0.001), and Microflash and Splitting needle (*p* = 0.013).

**Figure 2:**
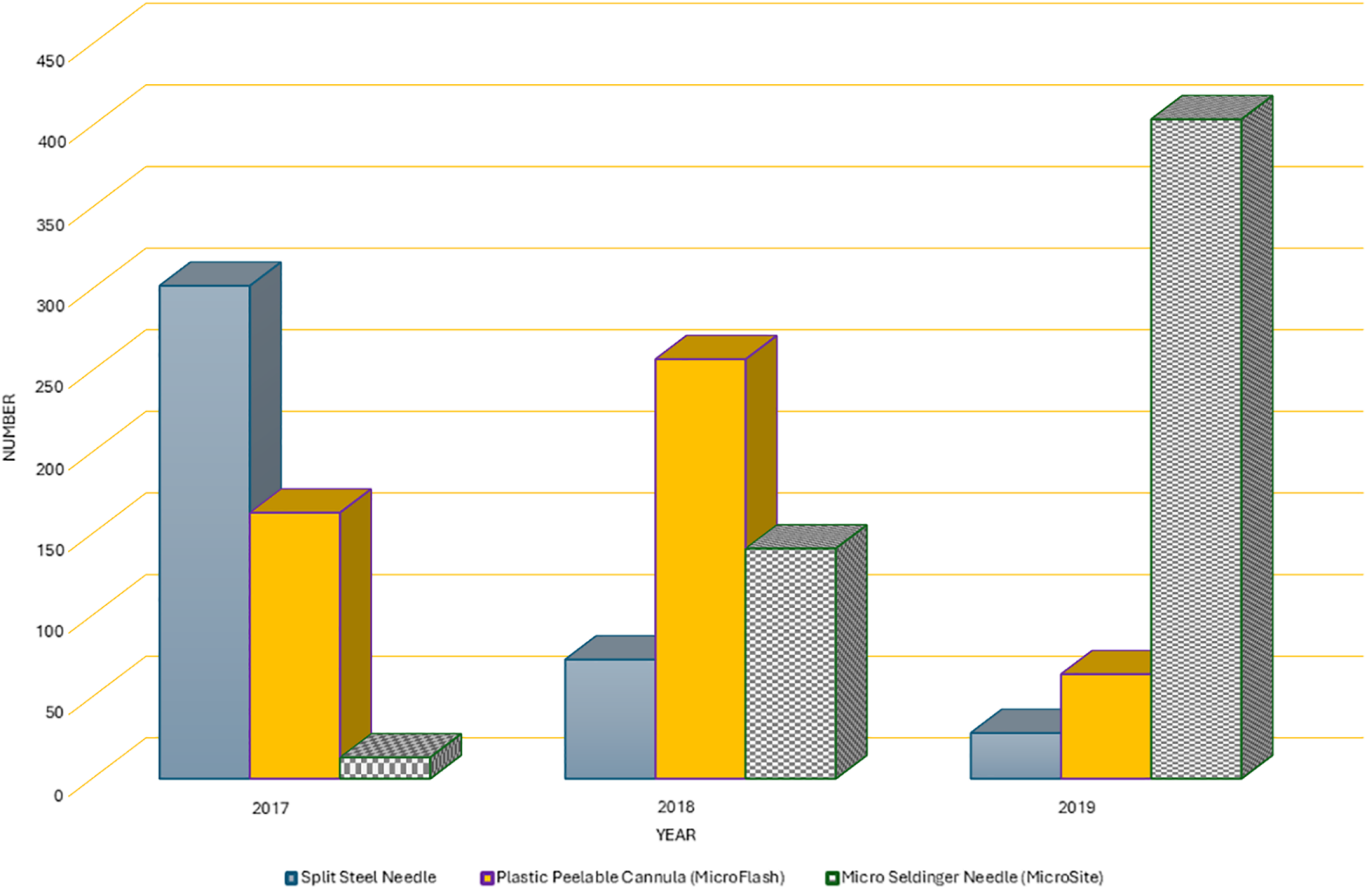
Insertion device use over 3 years.

Over the course of 3 years, there was a noticeable change in the pattern of usage of conventional insertion needles, as illustrated in Figure 1. In 2017, the Splitting needle was used in 63% of the cases (302 out of 478), but by 2019, it was utilized in only 6% of cases (28 out of 496). Simultaneously, the use of the Microflash insertion needle increased from 34% (163 out of 478) in the first year to 55% (257 out of 471) in the second year of the study. However, its use experienced a decline in the final study year, dropping to 13% (64 out of 496). Conversely, the use of the micro-MST kit exhibited a consistent upward trend in utilization, starting at 3% (13 out of 478) in 2017 and reaching 81% (404 out of 496) in 2019 (Figure 2).

### Complications

Comparing conventional insertion techniques with the micro-MST insertion kit, there was a significant difference in the reasons for catheter removal or successful end of therapy (Table 4). Detailed analyses per type of technique device used were in favor of Microsite devices/technique. The failure rate for micro-MST was 14% vs. Microflash with 19% and Splitting needle with 25%. While the dwell time was not significantly different, it demonstrated an improvement when the micro-MST kit was utilized.

**Table 4:** Reasons for the removal of the peripherally inserted central catheter (PICC).

	Totals	Conventional insertion (*n* = 781)	Micro-MST (*n* = 505)	*p-*value
Reason for removal				
1. Therapy completed	952 (74%)	551 (71%)	401 (79%)	0.001
2. Therapy failure^a^	242 (19%)	171 (22%)	71 (14%)	
Catheter-related complications^b^	100	72	28	
Maintenance-related complications^c^	142	99	43	
3. Administrative censoring^d^	92 (7%)	59 (7%)	33 (7%)	
Dwell time in days, mean ± SD		14.5 ± 8.7	14.9 ± 8.2	0.356
	Microflash *n* = 432	Splitting needle *n* = 349	Microsite *n* = 505	*p-*value
1. Therapy completed	316 (73%)	235 (67%)	401 (79%)	<0.001
2. Therapy failure^a^	84 (20%)	87 (25%)	71 (14%)	
Catheter-related complications^b^	31	41	28	
Maintenance-related complications^c^	53	46	43	
3. Administrative censoring^d^	32 (7%)	27 (8%)	33 (7%)	
Dwell time in days, mean ± SD	14.8 ± 8.4	14.1 ± 9.0	14.9 ± 8.2	0.343

SD, standard deviation.

^a^ Therapy failure = catheter-related and maintenance-related complications.

^b^ Catheter-related complications are defined as leaking, infiltration/extravasation, breakage of the catheter, and phlebitis.

^c^ Maintenance-related complications are defined as accidental removal, tip migration, and occlusion.

^d^ Administrative censoring = death and neonates transferred out to another facility.

### Central line-associated bloodstream infection

The definition of a CLABSI diagnosis lacks consistent clinical diagnostic criteria, and the use of that term can vary between settings (7). In this study setting, the definition of CLABSI and the calculation of its incidence rate aligned with the definitions of the US Centers for Disease Control and Prevention (CDC) (24). In essence, CLABSI was confirmed in the presence of a laboratory-confirmed bloodstream infection not associated with any other infection site and developed within 48 h of the central line insertion. The CLABSI rate is defined as the number of CLABSI infections per 1,000 central line days. The occurrences of CLABSI in this study are presented in Table 5. Micro-MST (Microsite) showed the lowest CLABSI rate (1.6%). Combined and individually conventional insertion techniques showed a 5.0% (4.9 and 5.1) CLABSI rate.

**Table 5:** Central line associated bloodstream infection (CLABSI).

		Conventional insertion (*n* = 781)		Micro-MST (*n* = 505)	*p-*value
CLABSI diagnosed^a^		39 (5.0%)		8 (1.6%)	0.001
Incidence^b^		3.45		1.06	
	Conventional (*n* = 781)		Micro-MST (*n* = 505)		
	Microflash (*n* = 432)	Splitting needle (*n* = 349)	Microsite (*n* = 505)		
CLABSI^a^	22 (5.1%)		17 (4.9%)	8 (1.6%)	0.006
Incidence^b^	3.44		3.47	1.06	

^a^ CLABSI is defined as a laboratory-confirmed bloodstream infection not associated with any other infection site and developing within 48 h of the central line insertion (21).

^b^ CLABSI incidence rate per 1,000 catheter days.

## Discussion

The neonates in the micro-MST group generally had a younger mean age compared to that in the conventional insertion group. There was no evidence that inserters chose the micro-MST for younger patients, and the gradual transition to greater use of the micro-MST approach over the study duration did not support this reasoning. It is possible that this observation might reflect greater utility and adherence to the VA route and device selection algorithm used in the study site (16, 21). This tool advocates for earlier selection of central vascular routes, such as PICC, when it is anticipated that intravenous therapy will be prolonged and would affect the age at which PICCs were inserted.

A detailed cost-effectiveness analysis of the two approaches to the PICC insertion was beyond the scope of this study. Some commentaries suggest that despite the additional cost of MST equipment, the overall costs are in favor of MST (6). Studies that attempted to analyze the economic effects of using MST for the PICC insertion suggest cost-neutral or slight economies (17, 19) However, when factors, such as the local pricing of MST equipment and staff salary, which vary between hospital facilities and internationally, and fewer insertion attempts are combined with the economic costs related to the local incidence of CLABSI, then the cost-effectiveness analysis becomes more complex. Further study using more sophisticated economic modeling is required to fully articulate the economic cost and benefits of MST.

### Insertion success

Both first time and overall PICC insertion success using the micro-MST approach was associated with increased insertion success compared to conventional techniques. However, in this study, the difference was not statistically significant. Wald et al. (18), using a specially modified bespoke MST insertion kit, reported a successful insertion in 14 of 16 cases. Other studies reported statistically significant improvements in insertion success. One example, Gibb and MacLeod et al. (17, 19) reported statistically significant improvements in insertion success when using a micro-MST kit similar to that used in this study compared to the splitting needle technique. However, these results need to be set in context. Gibb et al. and MacLeod et al. (17, 19) reported a comparatively lower first-attempt and overall insertion success rates for both techniques (MST 53% vs. 26% for steel splitting needle, overall, 72% vs. 40%) and more attempts to obtain VA (MST 2.5 vs. steel splitting needle 6.5) when compared to this study. This makes a direct comparison between these studies problematic.

### Complications

Two groups of non-infective complication were detailed in this study, that is, catheter related (leaking catheter, infiltration/extravasation, phlebitis, and catheter breakage) and maintenance related (tip migration, accidental catheter removal and occlusion). While these were defined in the database, they were not differentiated and therefore not available for further subgroup analysis. Consequently, we are unable to comment on the relative significance of these subgroups beyond the observation that, taken together, they did not statistically affect the comparative catheter dwell times.

The duration and frequency of clinical procedures, particularly those such as needlesticks associated with pain and discomfort, can have important implications for patient and parent welfare (25–27). The choice of catheter, insertion device, and insertion technique must be based upon a thorough assessment of the need for the prescribed therapy, blood vessel health and suitability for the intended catheter diameter and flow rate, possibility of DIVA (pre-existing or developing), and skill set of the inserter (6, 7, 15, 16), which have a bearing on patient experience, insertion success, and likelihood of complications. Reports (20) suggest that MST approaches involve more preparatory steps, which can be time consuming for those new to this technique and invariably require a period of training and practice for mastery. It might be that ongoing training, increased NeoVAT team cohesion, and the increased familiarity of staff with the micro-MST approach help explain the transition to greater use of this technique over the study time.

Speculatively, several interventions could have contributed to the high rates of first-attempt successful insertion and the lower incidence of catheter-related complications reported in this study. First, these might be related to the nature of the micro-MST approach and the intrinsic design characteristics of the kit, which are intended to inflict less trauma on the blood vessel endothelial wall. Second, these might be related to limiting the PICC insertion to a dedicated and highly trained group of staff (14). Third, it could be that the greater attention to vein preservation strategies and vein assessment, as advocated for by the routinely used 5-Rights mnemonic, was instrumental (4, 16, 21). For example, in this study, incorporating a system for routine systematic vein assessment could have aided insertion success and reduced complications by avoiding suboptimal veins. The embedding of this approach into routine practice might also offer an explanation for the greater selection of the saphenous vein in the micro-MST group as fewer instances of prior vessel use and compromise were likely to be encountered ensuring the vein suitability for the PICC insertion. However, further development of this tool and research to validate it and explore the implications in practice of its use are required.

### CLABSI

Reductions in CLABSI rates reported in this study using the micro-MST align with those reported in other neonatal studies (28, 29). Experiencing fewer episodes of skin breakage due to fewer attempts, reduced blood loss, and reduced trauma to the blood vessels due to the dilation technique and guidewire depth reported with MST might explain this finding (16–20). However, it is important to avoid overinterpreting this relationship. In this study, only a limited number of the multifactorial variables known to affect the incidence of CLABSI were included in the analysis. For example, previous studies highlighted the effects of adherence to preventative infection control care bundles (6, 7, 9, 11, 22, 23, 30–32) using closed-circuit infusion sets (32) and cyanoacrylate-based tissue adhesive to secure lines and seal insertion sites (22, 23), which were not analyzed here. Further large-scale interventional studies are required to separate the relationships between CLABSI and the PICC insertion technique in everyday clinical situations.

### Strengths and limitations

This study represents a valuable contribution to the limited body of research comparing various PICC insertion techniques specifically within the neonatal population. Despite its strengths, such as the inclusion of a sizable patient cohort, several methodological considerations warrant discussion.

The retrospective, single-center design employed in this study inherently introduces potential biases and limitations. Notably, the lack of randomization and the reliance on retrospective data collection may have introduced selection bias and confounding variables, thereby impacting the internal validity of the findings. Additionally, the retrospective nature of the study limits our ability to control all potential confounders, including changes in practice patterns over time. It is crucial to acknowledge the possibility of inserter variability and unrecorded shifts in procedural techniques or institutional protocols, which could have influenced the observed outcomes. However, the implementation of a dedicated team for PICC insertion, along with standardized training and educational programs, likely helped mitigate some of these potential sources of variability.

Despite the abovementioned limitations, this study provides valuable insights into the comparative effectiveness of different PICC insertion techniques in neonates. Future research endeavors should aim to address these limitations by employing prospective, multicenter study designs with stringent control measures. Such studies would not only validate these findings, but also offer a more nuanced understanding of optimal neonatal PICC insertion practices. Emphasis should be placed on the greater accounting of the multifaceted aspects of PICC insertion and use, as well as the potential impact of adherence to care bundles, promotion of comfort, provision of pain relief, utilization of tissue adhesive, and proactive removal strategies on the likelihood of CLABSI occurrence.

## Conclusion

Using the micro-MST kit for PICC insertion in neonates can improve first-attempt and overall insertion success rates and reduce catheter-related complications, leading to unplanned removal, therapy failure, and fewer cases of CLABSI. The micro-MST kit is a safe and effective approach to PICC insertion, and it has become popular among VA practitioners. However, further study is required to demonstrate the superiority of this insertion technique over conventional approaches.

## Data Availability

The datasets generated for this study are available on reasonable request to the corresponding author. Data requests should be made to Dr. Mohammad A. A. Bayoumi by email to moh.abdelwahab@hotmail.com.
